# Use of Low-Coverage, Large-Insert, Short-Read Data for Rapid and Accurate Generation of Enhanced-Quality Draft *Pseudomonas* Genome Sequences

**DOI:** 10.1371/journal.pone.0027199

**Published:** 2011-11-02

**Authors:** Heath E. O'Brien, Yunchen Gong, Pauline Fung, Pauline W. Wang, David S. Guttman

**Affiliations:** 1 Department of Cell and Systems Biology, University of Toronto, Toronto, Ontario, Canada; 2 Center for the Analysis of Genome Evolution & Function, University of Toronto, Toronto, Ontario, Canada; The University of Hong Kong, China

## Abstract

Next-generation genomic technology has both greatly accelerated the pace of genome research as well as increased our reliance on draft genome sequences. While groups such as the Genomics Standards Consortium have made strong efforts to promote genome standards there is a still a general lack of uniformity among published draft genomes, leading to challenges for downstream comparative analyses. This lack of uniformity is a particular problem when using standard draft genomes that frequently have large numbers of low-quality sequencing tracts. Here we present a proposal for an “enhanced-quality draft” genome that identifies at least 95% of the coding sequences, thereby effectively providing a full accounting of the genic component of the genome. Enhanced-quality draft genomes are easily attainable through a combination of small- and large-insert next-generation, paired-end sequencing. We illustrate the generation of an enhanced-quality draft genome by re-sequencing the plant pathogenic bacterium *Pseudomonas syringae* pv. phaseolicola 1448A (*Pph* 1448A), which has a published, closed genome sequence of 5.93 Mbp. We use a combination of Illumina paired-end and mate-pair sequencing, and surprisingly find that *de novo* assemblies with 100x paired-end coverage and mate-pair sequencing with as low as low as 2–5x coverage are substantially better than assemblies based on higher coverage. The rapid and low-cost generation of large numbers of enhanced-quality draft genome sequences will be of particular value for microbial diagnostics and biosecurity, which rely on precise discrimination of potentially dangerous clones from closely related benign strains.

## Introduction

The rapid development and wide-spread adoption of next-generation (next-gen) genomic technology provides unprecedented ability to generate genomic data, and consequently has dramatically increased our understanding of both the breadth and depth of biological diversity [1], [2]. Unfortunately, the nature of the technology has also dramatically increased our reliance on draft, rather than finished, genome sequences.

The Genomics Standards Consortium (GSC) and Human Microbiome Project Jumpstart Consortium designate a spectrum of genome sequence standards [3]:

- Standard Draft: minimally or unfiltered data, from any number of different sequencing platforms, that are assembled into contigs. This is the minimum standard for a submission to the public databases. Sequence of this quality will likely harbor many regions of poor quality and can be relatively incomplete. It may not always be possible to remove contaminating sequence data. Despite its shortcomings, Standard Draft is the least expensive to produce and still possesses useful information.
- High-Quality Draft: overall coverage representing at least 90% of the genome or target region. Efforts should be made to exclude contaminating sequences. This is still a draft assembly with little or no manual review of the product. Sequence errors and misassemblies are possible, with no implied order and orientation to contigs. This is appropriate for general assessment of gene content.
- Improved High-Quality Draft: additional work has been performed beyond the initial shotgun sequencing and High-Quality Draft assembly, by using either manual or automated methods. This should contain no discernable misassemblies and should have undergone some form of gap resolution to reduce the number of contigs and supercontigs (or scaffolds). Undetectable misassemblies are still possible, particularly in repetitive regions. Low-quality regions and potential base errors may also be present. This standard is normally adequate for comparison with other genomes.
- Finished: refers to the current gold standard; genome sequences with less than 1 error per 100,000 base pairs and where each replicon is assembled into a single contiguous sequence with a minimal number of possible exceptions commented in the submission record. All sequences are complete and have been reviewed and edited, all known misassemblies have been resolved, and repetitive sequences have been ordered and correctly assembled. Remaining exceptions to highly accurate sequence within the euchromatin are commented in the submission.

Despite these standards, there is a general lack of uniformity among published draft genomes, resulting in challenges for downstream comparative analyses. This is a particular problem for standard draft genomes, which frequently have reasonably large tracts that are at low quality, unresolved, or potential contaminants.

The logical resolution of this problem is to recommend that all genome sequencing projects be carried out to the high-quality draft level at a minimum. As discussed above, high-quality draft genomes represent at least 90% of the genome and have very little contamination. Unfortunately, this standard is fairly low for comparative analyses and fairly ambiguous since it does not explicitly address the representation of the genic component of the genome, which is clearly the component of primary interest to most researchers. Moving up to the next level of improved high-quality genome provides little additional help for two primary reasons. First, it again does not explicitly address the goal for the genic component of the genome. And second, it stipulates that there should be no discernable misassemblies, which is laudable, but nearly impossible to achieve or identify when performing *de novo* assembly on a divergent organism.

Here we propose the term “enhanced-quality draft” genome sequence to set a goal for genome sequences that effectively provide a full accounting of the genic component of the genome. An enhanced-quality draft genome would be one which identifies >95% of the coding sequences, although given the reality of repetitive sequences not all of these coding sequences would be complete. Further, an enhanced-quality draft genome would employ some manual or automated approach to increase scaffold length beyond what is typically achieved by short-insert paired-end sequencing, which cannot resolve assemblies through most repetitive elements. Ultimately, an enhanced-quality draft genome sequence would provide a near-complete account of a genome with the exception of full synteny information and repetitive elements, and therefore provide highly reliable and robust data for the vast majority of downstream comparative and functional analyses.

Hybrid sequencing approaches that combine Illumina and longer-read 454 sequencing have been proposed as a means to generate good-quality draft genomes [4]. Unfortunately, the per nucleotide cost of 454 sequencing and the cumbersome nature of working with two sequencing platforms decreases the utility of this approach. Here we present an alternative methodology that uses a combination of Illumina paired-end and mate-pair sequencing protocols (for short- and long-insert paired reads, respectively) to produce enhanced-quality draft genomes at minimal cost. On the Illumina platform, paired-end sequences are typically separated by two to four hundred base pairs, while mate-pair sequences are typically separated by four to six kilobase pairs. We illustrate the quality of the assemblies from this hybrid approach by sequencing the plant pathogenic bacterium *Pseudomonas syringae* pv. phaseolicola 1448A (*Pph* 1448A), which has a finished genome [5] of 5.93 Mbp, and discuss the power of this approach relative to that used in two other re-sequencing studies of *P. syringae* strains [4], [6].

## Methods

### Genomic DNA Extraction

Genomic DNAs were prepared from a modified protocol based on the Gentra Puregene Genomic DNA Purification Kit Instructions (Qiagen Canada, Toronto, ON), where all reagent volumes are doubled. For each strain, 800 µl of overnight cultures were centrifuged at 2000 x g for 5 minutes. The supernatant was removed and the pellet was resuspended in 600 µl of the Cell Lysis Solution per tube.12 µg RNase A was added per sample, mixed by inverting and incubated at 37°C for one hour. Samples were cooled to room temperature and 200 µl of Protein Precipitation Solution was added to the cell lysate and vortexed at high speed for 3 seconds. Samples were then centrifuged at 13,000 x g for 10 minutes and the supernatant was immediately transferred to a clean 1.5 ml tube and incubated on ice for 5 minutes. This 10-minute centrifugation was repeated until the supernatant was clear. DNA was precipitated by adding 600 µl of isopropanol to the clear supernatant and mixed by slow inversion. The DNA was spooled into 1 ml of 70% ethanol and centrifuged at 13,000 x g for 10 minutes. The supernatant was removed and the pellet was air-dried for approximately 15 minutes. The dried pellet was resuspended in 25 µl of TE buffer either by incubating at 4°C overnight or at 65°C for one hour followed by vortexing at low speed for 10 minutes.

### Library Preparation

Sequencing libraries were prepared according to standard Illumina protocols for paired-end and mate-pair sample preparation. DNA was sheared using the Covaris S-series (Woburn, MA). Mate-pair libraries were prepared using the mate-pair sample preparation kit from Illumina (San Diego, CA). However, the standard paired-end procedures of end repair, A-tailing, adapter ligation and PCR used enzymes from New England Biolabs (Ipswich, MA). Sequencing reads and assemblies are available from the CAGEF website (www.cagef.utoronto.ca).

### Genome Assembly

Read mapping and *de novo* assembly were carried out using the CLC Genomics Workbench version 4 (Århus, Denmark) using long-read assembly for paired-end data and short-read assembly for mate-pair data. Mismatch costs were set to 2 and indel costs were set to 3. Broad insert size ranges were selected to maximize the number of paired reads mapped (50–500 bp for paired-end reads, 500–8000 for mate-pair reads). *De novo* assemblies were also carried out using SOAPdenovo on the paired-end data with a range of kmer sizes from 57 to 63 and an average insert size inferred from the read mapping data. The minimum contig size cutoff for *de novo* assemblies was set to 200 bp. Contigs were scaffolded with the mate-pair data using the program SSPACE [7] using an insert size range of 4–6 kb (based on the observed distribution from the read mapping), minimum link number of 50 and maximum link ratio of 0.3. Assemblies and scaffolding were also carried out using randomly selected subsets of the read data to determine how much read depth is necessary for assembly. Scaffolding was carried out using the contigs obtained from the CLC assembly of all the paired-end reads, with the minimum link number reduced in proportion to the number of reads. For each read depth level, assemblies were carried out on three replicate samples of the reads.

Coverage statistics were determined from the mapping report and Single Nucleotide Polymorphisms (SNPs) and Insertion-Deletion polymorphisms (InDels) were detected using the tools in CLC. Contigs less than 3 kb in length were BLASTed against the *Pph*1448A reference sequence to check for contigs derived from contaminating sequence. Contigs and scaffolds were ordered relative to the *Pph*1448A reference sequence and aligned using the contig mover tool and the progressiveMauve algorithm in Mauve [8]. The number of mismatches and the cumulative length of insertions, gaps, and missing sequences between contigs were determined from the resultant alignment. The regions of the *Pph*1448A reference genome sequence corresponding to gaps in the alignment were BLASTed against the reference genome with an E-value cutoff of 10^−10^ to identify repetitive sequence, defined as regions with more than one hit (covering at least 80% of the sequence).

Open reading frames (ORFs) that match the annotated genes in the reference sequence were identified by comparing the amino acid translations of the genes against the contigs using tBLASTn. *Ab initio* gene predictions were carried out using the Rapid Annotation with Seed Technology (RAST) [9] and Integrated Microbial Genomes Expert Review (IMG/ER) [10] annotation pipelines. Gene predictions were compared to the reference sequence annotations by using BLASTp searches of translated amino acid sequences to identify matching genes, and pairwise alignments were generated using the Needle tool of the EMBOSS software suite [11]. Multi-copy genes were identified in the reference sequence by comparing amino acid translations using BLASTClust [12] using a cutoff of 90% overlap of the shorter sequence and 90% similarity.

## Results

Approximately eleven million clusters were generated for both the paired-end (short-insert) and mate-pair (long-insert) libraries. Paired-end sequencing was performed for 75 cycles per end, while the mate-pair sequencing was carried out for 38 cycles per end. Data-quality was high in both cases, with 82% and 87% of clusters passing the Illumina pipeline filter for the paired-end and mate-pair libraries respectively. When the filtered paired-end data were mapped against the *Pph* 1448A reference sequence, all but 465,000 of the 22 million reads mapped, with over 10 million of the 11 million pairs mapping within 500 bp of each other in the expected orientation, with the majority mapping between 150 and 300 bp of each other. All but one 8 bp region of the reference sequence adjacent to an IS element was covered by the reads, with an average read depth of 255X and maximum depth of 2095X. For the mate-pair data, 15.4 million reads mapped, with 5.4 million pairs mapping within 8 kb (majority between 4000 and 6000 bp). Every nucleotide position of the reference except 64 bp were covered, with an average depth of 95X and a maximum depth of 797X. The polymorphism detection tools in CLC identified 140 SNP positions, all but one of which were either a low depth site (<20X for alternate base) or were polymorphic, including the reference base and one or more other bases. There were also 23 indels detected. Of these, 18 had coverage of at least 20X and were present in more than 70% of the mapped reads.

The paired-end CLC assembles produced 465 contigs, with sizes ranging from 200 bp (the minimum cutoff) to 112,659 bp and a N50 of 27,408 bp (table 1). Read depth varied from 72x to 2955x, except for one 213 bp contig with depth of 12x. All of the short (<3 kb) contigs produced strong matches when BLASTed to the *Pph* 1448A reference sequence, indicating that no contaminating sequence was being included in the assembly. The total combined size of the contigs was 5,854,612, which is 258 kb smaller than the combined size of the *Pph* 1448A chromosome and plasmids. This difference is primarily due to repetitive regions being collapsed into a single high-coverage contig rather than missing single-copy genome. The best SOAPdenovo assembly was produced using a kmer size of 57, resulting in 165 scaffolds with N50 of 63,090 and a maximum scaffold size of 179,822. The total size was slightly smaller than the CLC assembly (5,426,173 bp).

**Table 1:** Assembly statistics for *P. syringae* pv. phaseolicola 1448A resequencing.

Assembler	Sequencing Depth a		Contig/Scaffold #	N50 (kb)	Coverage b, All ORFs		Coverage b, Single Copy ORFs		Mismatches
	PE	MP			100%	90%	100%	90%	
SOAPdenovo	250x		165	63	82%	88%	84%	91%	0.011%
SOAPdenovo/SSPACE	250x	100x	47	444	82%	88%	84%	91%	0.007%
SOAPdenovo/SSPACE	100x	5x	165	384	76%	93%	79%	96%	0.005%
CLC	250x		465	27	94%	96%	97%	98%	0.006%
CLC/SSPACE	250x	100x	129	507	94%	96%	97%	98%	0.010%
CLC/SSPACE	100x	5x	95	542	95%	97%	98%	99%	0.004%

a PE = paired-end sequencing; MP = mate-pair sequencing.

b Percent of all Open Reading Frames (ORFs) or only single copy ORFs that were complete (100%) or 90% reassembled.

When the contigs from either assembly method were aligned to the reference sequence using Mauve, they were aligned as a single local colinearity block except for the contigs containing the origins of replication for the chromosome and plasmids, which was inferred to have a rearrangement because the alignment algorithm assumes that the sequences are linear. The CLC contigs covered 96% of the reference sequence, including 99% of the non-repetitive sequence, while the SOAPdenovo contigs only covered 88% of the reference (90% of the non-repetitive sequence). Of the polymorphisms detected from read mapping, the one SNP and 8 of the 18 indels were found in the CLC assemblies. In addition, polymorphisms were identified at the same position or adjacent positions for 9 of the remaining 10 indels. The SOAPdenovo assembly contained 8 of the same polymorphisms as the CLC assembly, as well as the one indel that was detected in the read mapping but not in the CLC assembly. In addition to these polymorphisms, there are 192 novel sequences inserted in the CLC contigs, totaling 7.8 kb, 40 gaps totaling 21 kb, and 392 mismatches. The SOAPdenovo assembly had a similar number of insertions but had 820 gaps totaling 46 kb and 658 mismatches. This translates to an overall error rate (mismatches, novel sequence, and missing sequence within contigs) of 0.5% for the CLC assembly and 1% for the SOAPdenovo assembly.

Scaffolding of the CLC contigs using mate-pair data resulted in 129 scaffolds with an N50 of 506,721 (table 1). The scaffolds were also aligned as a single local colinearity block with the exception of the scaffolds containing the origins of the chromosome and the small plasmid. Scaffolding of the SOAPdenovo contigs produced 47 scaffolds with an N50 of 355,657. One of these scaffolds was a chimera that included contigs from both plasmids. The number of mismatches increased from 402 to 586 for the CLC assembly but decreased from 658 to 444 for the SOAPdenovo assembly. Thus, scaffolding dramatically increased contiguity with a relatively minor increase in error rates.

**Figure 1:**
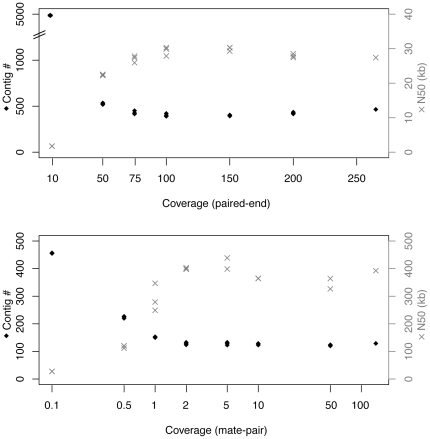
Relationship between quantity of sequence data (expressed as expected read depth) and assembly quality (number of contigs and N50) for subsamples of reads. A) *De novo* assemblies of 75×2 bp paired end reads (insert size 150–300 bp). B) Scaffolding of contigs using 38×2 bp mate-pair reads (insert size 4–6 kb). Three random subsets of reads were analyzed for each coverage level except the highest, which used all of the reads.

When CLC assemblies were done on random subsamples of the paired-end reads, assembly quality improved with increasing sequencing depth, but plateaued above 100X (figure 1a). A similar result was obtained for scaffolding with the mate pair data, though in the latter case, assembly quality plateaued between 2 and 5X (figure 1b). There was a lot of variability in scaffold N50 due to stochastic differences in which contigs were linked together, but the number of scaffolds was very consistent for each assembly above 2X. In both cases, slightly better assemblies were actually obtained with intermediate read depth, and a CLC assembly generated with 100X paired-end data and scaffolded with 5X mate-pair data resulted in 87 scaffolds and had a higher N50, more complete coverage of the reference sequence, and lower error rate than the assembly obtained with all the data (table 1). SOAPdenovo assemblies were more dependant on read depth, and assembly of 100X paired-end data resulted in a lower N50 and less coverage than assembly of all the data.

When the 5172 ORFs in the finished *Pph* 1448A genome were blasted against the CLC 250X contigs, 4945 ORFs (96%) were represented at >90% of their length, while 4876 of the ORFs (94%) were 100% represented (table 1). Of the 4982 single-copy genes, 4897 (98%) were identified, while 4851 (97%) were identical in sequence. A slightly larger number of ORFs was identified using the 100X CLC contigs while fewer ORFs were identified using the SOAPdenovo contigs. The gene callers from the two *ab initio* annotation pipelines that were used identified slightly fewer of the reference ORFs (4862 and 4722 for IMG and RAST respectively). In addition to finding more ORFs that match the reference, IMG also predicted slightly fewer ORFs that do not match (425 vs. 457).

## Discussion

The assemblies obtained for this study compare favorably with those obtained for other *P. syringae* strains using next-gen sequencing methods, including those based on long-read 454 technology. Using 250X paired-end Illumina data we obtained 465 contigs that could be combined using 11 million long-insert mate-pair reads into 129 scaffolds with an N50 of 507 kb. Previously published *P. syringae de novo* assemblies have obtained between 71 and 557 scaffolds with N50 values ranging from 26 to 317 kb [4], [13], [14], [15], [16], [17]. Our results also compare favorably to previous *P. syringae* resequencing projects which include a hybrid 26X Illumina single-read/∼7.5X 454 assembly of *Pto* DC3000 [4] and a 42X paired-end sequencing of *Psy* B728a [6]. The *Pto* DC3000 assembly resulted in scaffolds with an N50 of 90 kb which covered 99% of the reference, but was 6% larger than the reference (table 2). The best *Psy* B728a assembly had an N50 of 165 kb that covered 97.5% of the reference. Mismatch error rates were also lower than in previous studies: 0.010% for our study, compared to 0.027% for *Psy* B728a and 0.018% for *Pto* DC3000, though in the latter case, the rate was reduced to 0.014% by converting mismatches in repetitive regions to ambiguities.

**Table 2:** Comparison of *P. syringae* resequencing projects.

Strain	Sequencing Method & Depth a	Assembler	N50 (kb)	Completeness^b^	Complete ORFs^c^	Mismatches	Reference
*Pto*DC3000	Illumina (26x)/454 (2.5x/5xPE)	VCAKE/Newbler	90	99%	85%	0.018%	[4]
*Psy*B728a	Illumina (24xPE)	Velvet 0.718	165	97.5%	91%	0.027%	[6]
*Pph*1448A	Illumina (100xPE/5xMP)	CLC/SSPACE	542	98%	95%	0.004%	This study

a PE = paired-end sequencing; MP = mate-pair sequencing. If not specified then single read sequencing was performed.

b Percent of the single-copy portion of the reference genome present in contigs.

c Percent of all ORFs completely reassembled.

The high level of completeness and low error rate of our assembly mean that over 95% of annotated ORFs were complete and at least 2/3 of the ORF was present for 98% of single-copy genes. This rate is slightly higher than for the *Psy* B728a resequencing assembly (91%) [6], and considerably better than the *Pto* DC3000 resequencing assembly, where only 85% of the ORFs were 90% complete or better [4]. Of the two *ab initio* gene prediction methods used, the IMG pipeline out-performed RAST in terms of the number of predictions that match the reference annotation (4862 vs. 4722), the number of ORFs with matching start codons (3951 vs. 3682), and the number of ORFs that are not present in the reference annotation (425 vs. 457). However, this pipeline was still only able to predict 76% of ORFs in the reference annotation and only 92% of predicted genes were homologous to the reference genes. Clearly, a great deal of additional curation is needed to improve the results of *ab initio* gene predictions, even for prokaryotic organisms [18].

The quality of our enhanced-quality draft *Pph* 1448A assembly is due in part to the high sequencing depth that we obtained (250X paired end data, plus 100X mate pair data for scaffolding). This is almost an order of magnitude more data than was used for the *Psy* B728a sequencing project, and at least twice as high as was used for any other *P. syringae* sequencing projects to date. However, quite surprisingly, we were able to obtain an even better assembly by using a third of the paired-end data and 1/20th of the mate-pair data for scaffolding (figure 1), likely due to accumulation of reads with sequencing errors when excessive read depth is used. Our resampling test suggests that enhanced-quality draft genomes can be optimally produced with only 100x paired-end read depth and 2–5x mate-pair depth. These values were of course calculated based on the ∼6 Mbp genome of a pseudomonad, and will likely vary for bacterial isolates with substantially larger or smaller genomes and with a different abundance, makeup and distribution of repetitive elements. Nevertheless, it is clear from this study that the quality of the final assembly can be greatly improved with even a small amount of large insert data, and that sequencing depth is not positively associated with assembly quality.

Given current sequencing rates (80–125 cycles and >40 million quality clusters on the Illumina GAIIx platform), it is now possible to obtain enhanced-quality draft level *de novo* assemblies of large bacterial genomes for as many as 96 isolates on a single Illumina GAIIx flowcell. 120 cycle paired-end sequencing of 96 isolates across seven channels can yield >100x genome coverage, while mate-pair sequencing of the same isolates on the final channel of the flowcell will produce >15x coverage. These data should yield contig N50s of 10–30 kb, and scaffolds with N50 in the hundreds of kilobases. The costs incurred for such a project would currently be in the range of $250–$500 per bacterial genome sequence, although these prices will undoubtedly drop as sequencer throughput increases and as more efficient barcoding methods become available. Consequently, it is reasonable to assume that in the near future it will be possible to rapidly and reliably produce enhanced-quality draft quality genome sequences not only for scientific study, but also for applied purposes such as diagnostics and biosecurity monitoring.

## References

[1] Yahara T, Donoghue M, Zardoya R, Faith DP, Cracraft J (2010). Genetic diversity assessments in the century of genome science.. Curr Opin Environ Sustainability.

[2] O'Brien HE, Thakur S, Guttman DS (2011). Evolution of Plant Pathogenesis in *Pseudomonas syringae*: A Genomics Perspective.. Annu Rev Phytopathol.

[3] Chain PS, Grafham DV, Fulton RS, Fitzgerald MG, Hostetler J (2009). Genome project standards in a new era of sequencing.. Science.

[4] Reinhardt JA, Baltrus DA, Nishimura MT, Jeck WR, Jones CD (2009). De novo assembly using low-coverage short read sequence data from the rice pathogen *Pseudomonas syringae* pv. *oryzae*.. Genome Res.

[5] Joardar V, Lindeberg M, Jackson RW, Selengut J, Dodson R (2005). Whole-genome sequence analysis of *Pseudomonas syringae* pv. phaseolicola 1448A reveals divergence among pathovars in genes involved in virulence and transposition.. J Bacteriol.

[6] Farrer RA, Kemen E, Jones JDG, Studholme DJ (2009). *De novo* assembly of the *Pseudomonas syringae* pv. *syringae* B728a genome using Illumina/Solexa short sequence reads.. FEMS Microbiol Lett.

[7] Boetzer M, Henkel CV, Jansen HJ, Butler D, Pirovano W (2011). Scaffolding pre-assembled contigs using SSPACE.. Bioinformatics (Oxford, England).

[8] Darling AE, Mau B, Perna NT (2010). progressiveMauve: multiple genome alignment with gene gain, loss and rearrangement.. PLoS One.

[9] Aziz RK, Bartels D, Best AA, DeJongh M, Disz T (2008). The RAST server: rapid annotations using subsystems technology.. BMC Bioinformatics.

[10] Markowitz VM, Korzeniewski F, Palaniappan K, Szeto E, Werner G (2006). The integrated microbial genomes (IMG) system.. Nucleic Acids Res.

[11] Rice P, Longden I, Bleasby A (2000). EMBOSS: the European Molecular Biology Open Software Suite.. Trends Genet.

[12] Dondoshansky I, Wolf Y (2000). BLASTCLUST - BLAST score-based single-linkage clustering.

[13] Almeida NF, Yan S, Lindeberg M, Studholme DJ, Schneider DJ (2009). A draft genome sequence of *Pseudomonas syringae* pv. tomato T1 reveals a type III effector repertoire significantly divergent from that of *Pseudomonas syringae* pv. tomato DC3000.. Mol Plant-Microbe Interact.

[14] Green S, Studholme DJ, Laue BE, Dorati F, Lovell H (2010). Comparative genome analysis provides insights into the evolution and adaptation of *Pseudomonas syringae* pv. *aesculi* on *Aesculus hippocastanum*.. PLoS One.

[15] Qi M, Wang D, Bradley CA, Zhao Y (2011). Genome Sequence Analyses of *Pseudomonas savastanoi* pv. glycinea and Subtractive Hybridization-Based Comparative Genomics with Nine Pseudomonads.. PLoS One.

[16] Rodríguez-Palenzuela P, Matas IM, Murillo J, López-Solanilla E, Bardaji L (2010). Annotation and overview of the *Pseudomonas savastanoi* pv. savastanoi NCPPB 3335 draft genome reveals the virulence gene complement of a tumour-inducing pathogen of woody hosts.. Environ Microbiol.

[17] Studholme DJ, Gimenez Ibanez S, MacLean D, Dangl JL, Chang JH (2009). A draft genome sequence and functional screen reveals the repertoire of type III secreted proteins of *Pseudonomas syringae* pathovar *tabaci* 11528.. BMC Genomics.

[18] Bakke P, Carney N, Deloache W, Gearing M, Ingvorsen K (2009). Evaluation of Three Automated Genome Annotations for *Halorhabdus utahensis*.. PLoS One.

